# Excellent outcomes following repair of Grade I–II isolated posterolateral corner injury and meniscal instability using an all‐inside, arthroscopic technique

**DOI:** 10.1002/jeo2.70462

**Published:** 2025-10-30

**Authors:** George Komnos, Vasileios Mitrousias, Victoria Duthon, Jacques Menetrey

**Affiliations:** ^1^ Centre de médecine du sport et de l'exercice, Swiss Olympic Medical Center, Hirslanden Clinique La Colline Geneva Switzerland; ^2^ Department of Orthopaedic Surgery & Musculoskeletal Trauma University Hospital of Larissa, School of Health Sciences, University of Thessaly Larissa Greece; ^3^ Service de chirurgie orthopédique University Hospital of Geneva Geneva Switzerland

**Keywords:** arthroscopic technique, knee, lateral meniscus instability, posterolateral corner injury, posterolateral instability

## Abstract

**Purpose:**

Injuries of the posterolateral corner (PLC) of the knee can lead to chronic rotational instability, pain and functional impairment. Isolated PLC injuries are commonly combined with lateral meniscus instability and are often overlooked, causing persistent symptoms and instability in patients. The purpose of this study was to introduce an all‐inside, arthroscopic technique for treating isolated, Grade I–II PLC injuries combined with lateral meniscus instability and report its clinical outcomes and failure rates.

**Methods:**

A total of 52 patients who had been diagnosed with isolated Grade I–II PLC injury with concomitant lateral meniscus instability, and were treated between 2014 and 2019 with the proposed technique, were included. Demographic, preoperative, intraoperative and postoperative data were collected and reviewed. Patient‐reported outcomes included a visual analogue scale (VAS) assessing pain, the International Knee Documentation Committee Subjective Knee Function (IKDC‐SKF) score, the Tegner Activity Scale and a patient‐acceptable symptom state (PASS). At follow‐up, all patients were clinically evaluated by an independent, fellowship‐trained orthopaedic surgeon, using the grinding test, the dial test, the posterolateral drawer test, the varus stress test and the McMurray test. Failure was defined as a negative PASS response, the need for revision surgery or the persistence of rotational instability as demonstrated by clinical tests.

**Results:**

At a mean follow‐up of 28.1 months, 98% of the patients declared satisfied with the procedure. Rotational instability, as assessed by the grinding test, dial test, posterolateral drawer test and varus stress test, showed significant improvement in 51 out of 52 patients (*p* < 0.001). The failure rate was 1.92% (1 patient), and no complications were noticed. The mean postoperative VAS score for pain was 1.2 (±0.8), the mean postoperative IKDC score was 86.3 (±5.5) and the mean postoperative Tegner Activity Scale score was 7.7 (4–10). In total, 76.9% of the patients returned to their pre‐injury activity levels.

**Conclusion:**

All‐inside, arthroscopic repair of subtle, isolated PLC injuries combined with lateral meniscus instability can result in favourable clinical outcomes, high patient satisfaction and excellent return‐to‐sport rates. High suspicion and meticulous clinical examination are required to successfully diagnose these injuries, which are often missed during the initial presentation.

**Level of Evidence:**

Level III.

**Abbreviations**
ACL: anterior cruciate ligament
BMI: body mass index
IKDC‐SKF: International Knee Documentation Committee Subjective Knee Function
LCL: lateral collateral ligament
MCL: medial collateral ligament
PASS: patient‐acceptable symptom state
PFL: popliteofibular ligament
PLC: posterolateral corner
PMFs: popliteomeniscal fascicles
PT: popliteus tendon
VAS: visual analogue scale

## INTRODUCTION

The typical mechanism of posterolateral corner (PLC) injury includes a direct varus stress at the knee or a non‐contact hyperextension and external rotation injury. Consequently, PLC injuries usually occur in combination with cruciate ligament rupture or are part of a multiligament injury [30]. However, subtle, isolated PLC injuries can also occur, sometimes following even minor knee sprains, especially in the athletic population [6, 17, 18, 19]. These injuries are often overlooked or diagnosed with a delay, since they are not easily identified in the magnetic resonance imaging (MRI) and require increased awareness and specific knee clinical tests [33, 34]. According to the available classifications, these injuries can be classified as Grade I–Grade II, based on varus and rotational instability of the knee in full extension [22], or as type B, with the knee at 30° of flexion [10].

It has been recently reported that concomitant lateral meniscus instability is usually present in such PLC injuries [19]. Anatomically, this can be explained by the delicate nature of the popliteus hiatus. Between the outer edge of the lateral meniscus and the popliteus tendon (PT), there are three small synovial ligamentous structures, known as the postero‐superior, postero‐inferior and anterior popliteomeniscal fascicles (PMFs), and a thin, fibrous band, the meniscofibular ligament [20, 36]. Traumatic disruption of the PMFs and/or the meniscofibular ligament during a subtle PLC injury provokes a concomitant lateral meniscus instability and clinically manifests as pain in the lateral compartment of the knee and mechanical symptoms such as catching, locking and giving way.

Management of these injuries is challenging, since there are sparse data regarding the conservative and surgical options in the literature. In a recent consensus on PLC injuries, it is stated that: ‘acute PLC injuries should be addressed surgically within 2‐3 weeks following injury’ [7]. But while surgical management of Grade III injuries has well‐documented results [17, 30, 31, 46], there are no data on the surgical treatment of isolated Grade I–II injuries. Conservative management of such low‐grade PLC injuries may have positive outcomes, but can be associated with persistent instability, while there is no evidence of the long‐term effects of the injury on the knee joint [23, 25]. Based on the latter, surgical treatment of low‐grade injuries should be considered, especially in the athletic population, aiming to restore knee stability and help patients achieve a complete and symptom‐free return to pre‐injury activity levels [6, 35].

In this way, the goal of this study is to introduce a new, arthroscopic, all‐inside technique for managing isolated, subtle, low‐grade PLC injuries with concomitant lateral meniscus instability and to report the short‐term clinical outcomes on a series of treated patients. The hypothesis of this study is that the proposed arthroscopic repair can be an effective treatment option, providing satisfactory clinical outcomes.

## MATERIALS AND METHODS

### Ethical approval and study design

The study was approved by the Commission Cantonale d'Ethique de la Recherche sur l'être humain de Gevève (Decision No. 2865). The research was performed in accordance with the Declaration of Helsinki Ethical Principles for Medical Research involving Human Subjects as revised in 2008, and all patients were informed about the study's goals and consented to include their data.

This is a single‐centre, single‐surgeon retrospective case series. Between 2015 and 2019, 204 patients underwent surgical treatment for PLC injuries at our institution. Of these, 71 patients were diagnosed with Hughston Grade I/II or Fanelli–Larson Type B isolated PLC injury and treated with the proposed, all‐inside, arthroscopic technique.

### Inclusion and exclusion criteria

Any primary Hughston Grade I/II or Fanelli–Larson Type B isolated PLC injury associated with lateral meniscus instability and treated arthroscopically with the proposed, all‐inside technique was the main inclusion criterion. Exclusion criteria were the following: open repair technique, associated cruciate ligament injuries, associated medial collateral ligament (MCL) injury, concomitant meniscal tears, concomitant chondral lesions > Outerbridge Grade II, history of ipsilateral knee surgery, age < 18 years, body mass index (BMI) > 40 and follow‐up < 1 year.

### Imaging evaluation

Preoperatively, weight‐bearing anteroposterior view radiographs as well as Rosenberg, Merchant and lateral views were performed in all patients. An MRI evaluation was also available for all patients. MRI assessment was performed by the treating physician according to recent guidelines [36].

### Intraoperative evaluation and surgical technique

**Figure 1:**
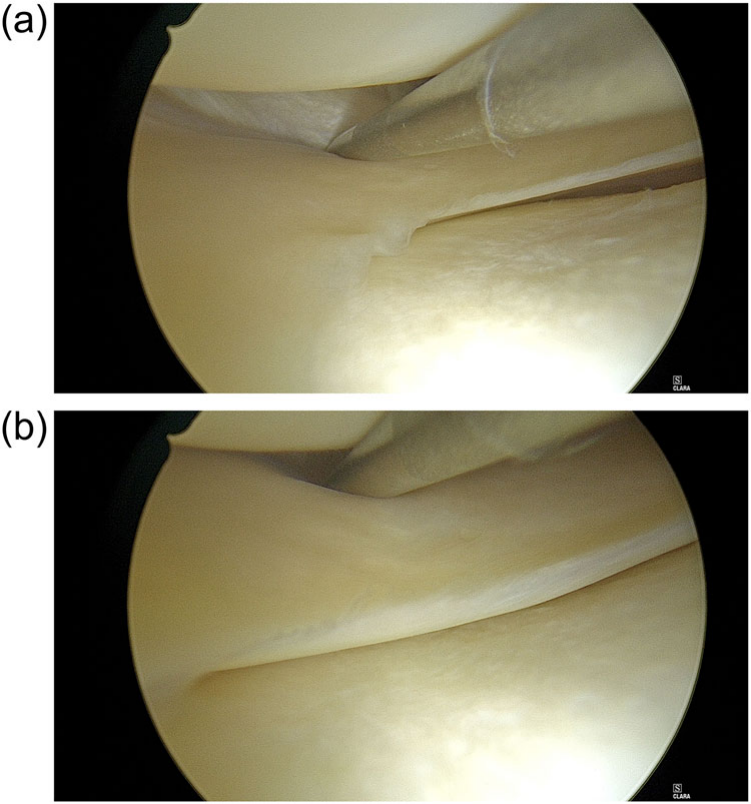
(a) Lateral meniscus position before pulling with the probe. (b) Lateral meniscus position after pulling with the probe. The lateral meniscus is subluxated into the middle of the lateral femorotibial compartment, and the probing stability test is considered positive.

**Figure 2:**
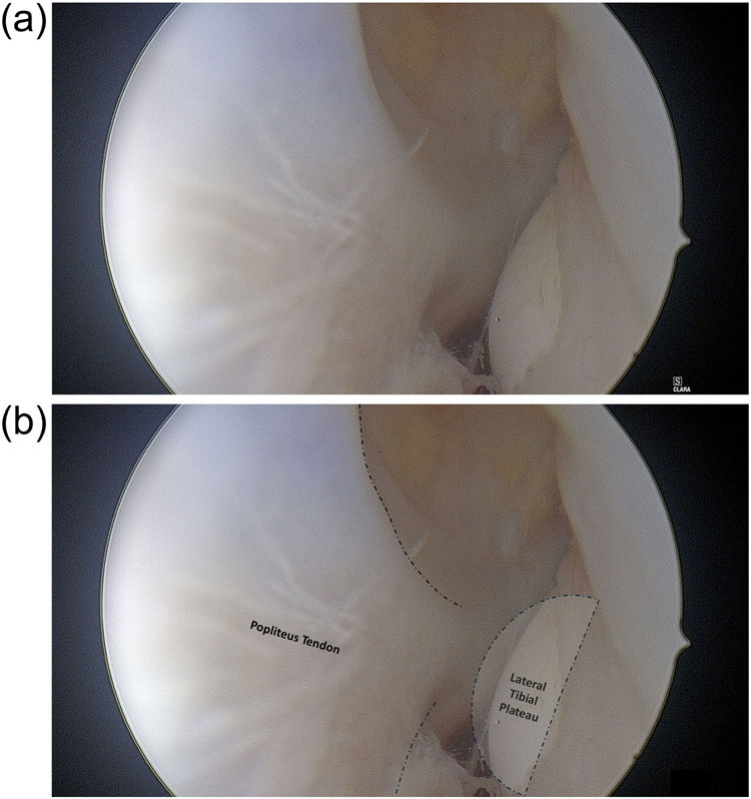
Arthroscopic image of the popliteal hiatus. (a) Without annotation and (b) with annotation. The cartilage border of the lateral tibial plateau appears as a large crescent shape. The ‘crescent moon sign’, which is a sign of the meniscofibular ligament rupture, is considered positive.

A typical diagnostic arthroscopy was performed by the treating physician. Since low‐grade PLC injuries have been associated with lateral meniscus instability, a meticulous evaluation of the lateral meniscus took place [18]. The lateral drive through test and the probing stability test (Figure 1a,b), assessing the integrity of the popliteus hiatus, the PMFs, the meniscofibular ligament and the displacement of the lateral meniscus were performed in all patients [11, 12, 13, 18]. Moreover, the presence of the ‘crescent moon’ sign (Figure 2a,b), pathognomonic of severe meniscus instability, was recorded when present [18]. The lateral meniscus probing stability test was considered positive when the posterior and intermediate horn could be displaced anteriorly by more than 50% of its width using a probe [38].

**Figure 3:**
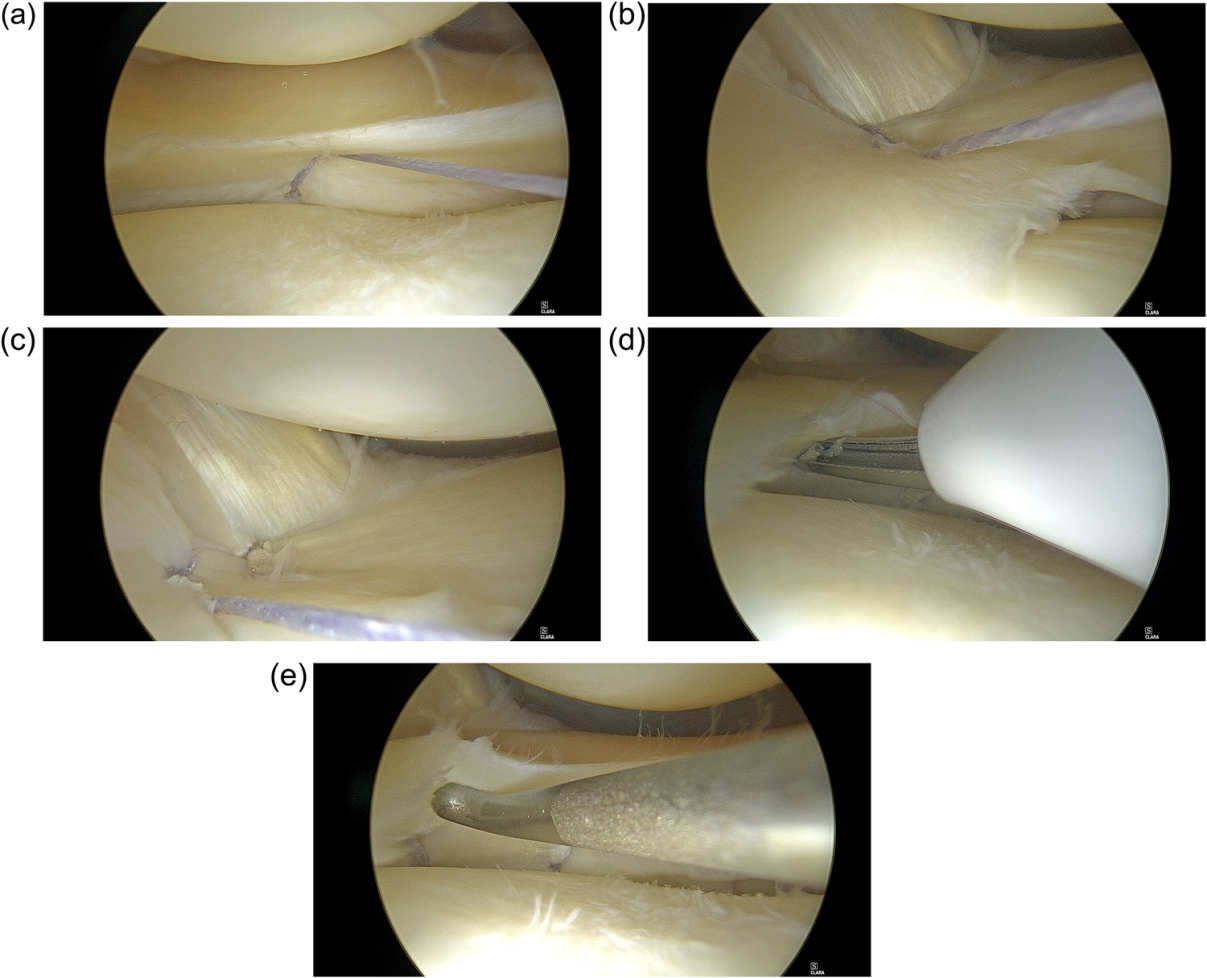
To perform the proposed technique, usually three all‐inside sutures are used. (a) The first suture is a vertical suture involving the inferior meniscal rim and the inferior part of the capsule. (b) The second suture is a vertical suture involving the superior meniscal rim and the most posterior part of the popliteal tendon. (c) The third suture is a vertical suture involving the superior meniscal rim and the capsule located anterior to the popliteal tendon. (d, e) A fourth suture can be added at the inferior aspect of meniscus, to better fix the meniscofibular ligament. This suture is placed inferiorly, about 3 mm from the meniscal rim and then goes down to the meniscofibular ligament. This is a vertical and inferior suture.

The proposed arthroscopic, all‐inside technique was performed by typical anterolateral and anteromedial portals with the knee placed in the figure of four position. The average duration of the procedure was 30 min. The goal of the technique was to stabilise the lateral meniscus and tighten the injured PLC structures. Rasping of the meniscus and adjacent synovium to stimulate healing was performed at first. Then, all‐inside sutures were placed, stabilising the lateral meniscus to the PT and the joint capsule. In this case series, the Truespan meniscal repair system was used (Johnson & Johnson, New Brunswick, NJ). The number of sutures was determined based on the grade of instability and the size of the knee. In all patients, at least three all‐inside sutures were placed. In cases where lateral meniscus instability extended to the posterior horn, one or two more sutures were added. The first suture was placed posteriorly to the popliteus hiatus, through the loose meniscotibial attachments and inferior peripheral rim of the lateral meniscus. This was a vertical suture involving approximately 5 mm of the meniscal rim and the most inferior part of the capsule (Figure 3a). The second suture was placed posteriorly to the popliteus hiatus, through the loose popliteofibular attachments and superior peripheral rim of the lateral meniscus. This was a vertical suture involving approximately 5 mm of the meniscal rim and the most posterior part of the popliteal tendon (Figure 3b). The third suture was placed anteriorly to the popliteus hiatus, through the loose popliteofibular attachments and superior peripheral rim of the lateral meniscus. This was a vertical suture involving approximately 5 mm of the meniscal rim and the capsule located anterior to the popliteal tendon (Figure 3c). A fourth suture can be added at the inferior aspect of the meniscus. To better fix the meniscofibular ligament, the suture is placed inferiorly, about 3 mm from the rim and then goes down to the meniscofibular ligament. This is a vertical and inferior suture (Figure 3d).

### Preoperative and postoperatvie clinical evaluation

All patients underwent a complete preoperative knee examination by the treating physician, assessing the integrity of the cruciate ligaments, the collateral ligaments, the PLC and the menisci. Postoperative evaluation was performed by an independent, fellowship‐trained orthopaedic knee surgeon.

Based on the inclusion criteria, patients of this case series had normal findings on the tests assessing the anterior cruciate ligament (ACL), the PCL, the MCL and the medial meniscus, but positive findings on the tests assessing the integrity of the PLC and the lateral meniscus.

Assessment of the PLC injury preoperatively and of the repair postoperatively included the following clinical tests: the varus stress test, the posterolateral drawer test, the dial test, the grinding test, otherwise known as the posterolateral rotatory drawer manoeuver and the McMurray test [3, 8, 27, 28].

The varus stress test was performed at 0° and 30° of flexion. The results were compared with the contralateral knee and categorised as Grade I for a 0–5 mm increase of gapping, Grade II for a 5–10 mm increase, and Grade III for a greater than 10 mm increase according to Hughston et al. [21]. The dial test was performed at 30° and 90° of flexion. Measurements were performed using a goniometer. The test was considered positive if the external rotation was increased more than 10° compared to the contralateral side. The grinding test was considered positive when posterior subluxation of the lateral tibial plateau that caused the anterior edge of the tibial plateau to be posteriorised in relation to the anterior edge of the lateral femoral condyle was noticed [3]. Similarly, the posterolateral drawer test was considered positive when posterolateral tibial subluxation was noticed [27].

### Patient‐reported outcome measures

Patient‐reported outcomes were assessed by the following questionnaires: a visual analogue scale (VAS) assessing pain (0: No pain; 10: Worst possible pain), the International Knee Documentation Committee Subjective Knee Function (IKDC‐SKF) score, the Tegner Activity Scale and a patient‐acceptable symptom state (PASS). The PASS specifically asked patients: ‘Taking into account your level of pain, your daily life activities and you sport participation limitations and restrictions, do you consider the current state of your knee satisfactory?’, through a binary answer, ‘yes’ or ‘no’. Failure was defined as a negative PASS response, the need for revision surgery or the persistence of rotational instability as demonstrated by clinical tests.

### Rehabilitation

After surgery, a knee brace permitting a range of motion 0°−90° for 6 weeks was used in all patients. Partial weight bearing up to 20 kg for 4 weeks was also advised. Full weight bearing and cycling were allowed as early as Week 7, jogging was allowed as early as Week 13 and the decision to return‐to‐sports was taken individualised based on the progression of each patient. The average duration from surgery to return‐to‐sports was 4.5 months.

### Statistical analysis

Statistical analysis was performed using the SPSS software (version 22.0). Descriptive statistics included mean, standard deviation, median, minimum and maximum values for continuous variables. Categorical variables were summarised using frequency counts and percentages. Comparisons were made using the chi‐square test.

## RESULTS

In total, 54 patients met the criteria to be included in the study. Two patients were lost during follow‐up. The final sample of this study included 52 patients (27♂:25♀). Mean age was 34.1 years (18–57). The mean time between injury and surgery was 13.2 months (1–92). The mean follow‐up was 28.1 (16‐44; SD ±7) months.

Intraoperative assessment revealed a positive lateral drive‐through test in all patients (100%). Preoperative and postoperative clinical assessment and results of the varus stress test, the posterolateral drawer test, the dial test, the grinding test and the McMurray test are presented in Table 1. No infections and no implant‐related complications were noticed. The reoperation rate was 1.9% since only one patient had to be treated with open repair for persisting instability, 9 months after initial surgery.

**Table 1:** Preoperative and postoperative clinical assessment of the treated patients.

Clinical tests	Positive preoperatively	Positive postoperatively	*p* value
Varus stress test 0°	0/52 (0%)	0/52 (0%)	‐
Varus stress test 30°	Grade I 27/52 (51.9%) Grade II 25/52 (48.1%)	Grade I 1/52 (1.9%) Grade II 0/52 (0%)	*p* < 0.0001
Posterolateral drawer test	52/52 (100%)	1/52 (1.9%)	*p* < 0.0001
Dial test 30°	52/52 (100%)	1/52 (1.9%)	*p* < 0.0001
Dial test 90°	50/52 (96.1%)	1/52 (1.9%)	*p* < 0.0001
Grinding test/posterolateral rotatory drawer manoeuver	52/52 (100%)	1/52 (1.9%)	*p* < 0.0001
McMurray test	49/52 (94.2%)	0/52 (0%)	*p* < 0.0001

The PASS statement was positive for 98% of the patients (51/52). The mean postoperative IKDC score was 86.3 (±5.5). The mean postoperative VAS score for pain was 1.2 (±0.8). The mean Tegner score postoperatively was 7.7 (4–10), and 76.9% of the patients returned to their pre‐injury activity levels.

## DISCUSSION

The main finding of this study is that the proposed, arthroscopic, all‐inside technique can successfully treat low‐grade, PLC injuries associated with lateral meniscus instability, since clinical restoration of the rotational instability was confirmed in the vast majority of the patients (98%) assessed during follow‐up. Second, the excellent outcomes on the VAS for pain, the IKDC score and the Tegner scale confirm the efficacy of the proposed technique, which allows patients to return, pain‐free, to their pre‐injury activity levels. To our knowledge, this is the first study to report on clinical outcomes of arthroscopic operative treatment following Grade I–II or type B PLC injury.

Another interesting finding of the study is the confirmation of the difficulty to diagnose these low‐grade PLC injuries. The mean time from injury to surgery for the examined sample was 13.2 months. All patients were presented for consultation in a specialised knee clinic, following failed conservative treatment with persistent symptoms of knee pain and instability. This can be partially explained by the fact that in the absence of an ACL or PCL injury, the PLC is often neglected [33, 34]. Moreover, identification of the smaller structures of the PLC is traditionally difficult on imaging, and thus Grade I/II or type B injuries can be easily missed, leading to chronic instability [5]. Similarly, identification of the concomitant lateral meniscal instability is also challenging. Interestingly, regarding the status of the lateral meniscus, a normal MRI does not rule out meniscal instability, and simultaneously, an abnormal MRI does not necessarily correspond to an arthroscopically unstable lateral meniscus [37, 42, 44]. Consequently, clinicians cannot fully rely on imaging to diagnose this specific condition, and clinical examination is far more important. High suspicion and a meticulous clinical assessment are the keys for diagnosis.

Nowadays, anatomy and biomechanics of the PLC are well‐defined, and thus various techniques including repair or reconstruction of the PLC have been proposed in the literature [4, 26, 43, 46, 47]. In a recent systematic review, 10 arthroscopic techniques for PLC injuries treatment have been identified [45]. A detailed analysis of these techniques reveals the arthroscopic equivalents of well‐established anatomical reconstructions for the PLC. These include the arthroscopic adaptation of the Arciero anatomical PLC reconstruction with various modifications [2, 15, 29] and the arthroscopic equivalent of the LaPrade technique [24]. Additionally, various arthroscopic approaches have been described for reconstructing the PT [11, 16] and the popliteofibular ligament (PFL) [39]. Among the available techniques, the approaches proposed by Ohnishi et al. [32] and Hermanowicz et al. [20] differ in their underlying biomechanical rationale. Hermanowicz et al. introduced an arthroscopic PT tenodesis technique, in which the PT is sutured arthroscopically and secured within a tibial tunnel. This technique aims to restore external rotational instability by recreating a static stabiliser [20]. In another effort, Ohnishi et al. developed a method focused on tightening the posterolateral capsule. Their approach employs an anchor at the tibial plateau to reattach the capsule and lateral meniscus, thereby enhancing rotational stability [32]. While the exact indications have not been well described, all these techniques have been mainly developed to address high‐grade PLC instability, usually in association with posterior cruciate ligament reconstruction [45]. However, the techniques proposed by Hermanowicz et al. and Ohnishi et al. could be used to treat low‐grade, isolated, PLC injuries, as described in this study's sample. Therefore, analysing their outcomes alongside the findings of the present study may provide valuable insights.

While the most important factor when comparing the above techniques with the one introduced by the present study would be the clinical outcomes, unfortunately, most authors have not (yet) published short‐ or long‐term results of patients who were treated accordingly. With regard to technical issues, the presented technique is simpler, since it does not require an accessory portal, a drilling guide, a suture‐passer or anchors to be performed. Additionally, the proposed surgical technique anatomically has a double effect: first, it tightens the elongated anatomical structures of the PLC, and second, it stabilises the lateral meniscus by restoring the anatomy of the popliteus hiatus. This is very important, since while the presence of a lateral meniscus instability in combination with low‐grade PLC injuries has been reported in the literature, it is not addressed by the already published techniques [18, 45]. To date, various all‐inside techniques have been proposed to treat lateral meniscus instability [1, 9, 40, 41]. However, their effect on the rotational stability of the knee is not reported, since the relationship between the PLC injury and the lateral meniscus instability is poorly recognised and usually remains undiagnosed. The favourable outcomes of the present study underline that not only the lateral meniscus hypermobility but also the rotational instability due to subtle PLC injuries can be treated arthroscopically.

Patient‐reported outcomes of patients treated for Grade I/II or type B PLC injuries are missing from the literature, as well as their return‐to‐sport rates. Analysing the results of high‐grade injuries, in a recent review of twenty‐nine studies assessing outcomes of open repair or reconstruction of PLC injuries associated with ACL or PCL injury, the mean IKDC score ranged from 59.8 to 87.3 for acute and 64 to 91.9 for chronic injuries, results comparable to the mean score of 86.4 reported by the present study's sample [46]. Obviously, the proposed arthroscopic technique shares the same advantages of all arthroscopic approaches in comparison to open procedures, such as reduced infection rates, less postoperative pain, faster rehabilitation, less scar tissue formation and a more aesthetical result. While in open techniques, failure rates are highly variable, ranging from 0% to 15% for reconstruction and 0% to 40% for repair [14, 46], in this case series, only one failure was reported, a fact that underlines the efficacy of the proposed, all‐inside technique. In terms of return‐to‐sports, results from open repair or reconstruction provide very high rates ranging from 94% to 100% [14]. However, the rates of return to the same level of sports range between 46% and 71% and are lower compared to those reported in the present sample, given the lower grade of PLC injury and the arthroscopic, all‐inside approach used. Regarding the timeline for returning to sports, an expert consensus statement recommended that after an isolated PLC reconstruction, return‐to‐sport should not be advised before 9 months [7]. However, it is important to emphasise individualising decisions for each patient, based on his/her concomitant injuries, the type of repair and the overall progress of the rehabilitation.

Finally, it is noteworthy that a direct comparison with conservative management outcomes remains challenging due to the limited number of relevant studies, most of which date back to the 1990s. The studies by Kannus et al. and Krukhaug et al. report generally favourable outcomes with conservative treatment; however, they also highlight a high incidence of persistent laxity [23, 25]. These observations are consistent with the authors' clinical experience, as a considerable number of patients in the present cohort sought a second opinion following unsuccessful conservative management, often presenting with residual instability and knee pain. While comparative studies are warranted, the excellent clinical outcomes of the present cohort suggest that surgical intervention may offer superior results, particularly in highly active individuals.

### Limitations

The present study has several limitations. First, patient assessments were conducted by a single surgeon, making clinical tests and instability evaluations potentially susceptible to subjectivity bias. However, it is important to note that the assessments were performed by an independent surgeon who was not involved in the surgical procedures. Second, at the time of the study and to date, no widely accepted method or device exists for the precise measurement of knee rotational laxity. Consequently, the decision to proceed with surgery was based on clinical findings, and the postoperative assessment of rotational laxity relied on the listed clinical tests. Third, the absence of preoperative IKDC scores precluded a direct comparison between preoperative and postoperative scores. Finally, the retrospective design of the study is also susceptible to selection and attrition bias.

## CONCLUSION

All‐inside, arthroscopic repair of subtle, isolated PLC injuries combined with lateral meniscus instability can result in favourable clinical outcomes, high satisfaction and excellent return‐to‐sport rates. High suspicion and meticulous clinical examination are required for successful identification of these injuries, which are often missed during initial presentation.

## AUTHOR CONTRIBUTIONS

*Conceptualisation*: George Komnos, Vasileios Mitrousias, Victoria Duthon and Jacques Menetrey. *Methodology*: George Komnos and Jacques Menetrey. *Resources*: George Komnos, Vasileios Mitrousias, Victoria Duthon and Jacques Menetrey. *Software*: George Komnos and Vasileios Mitrousias. *Writing—original draft preparation*: George Komnos and Vasileios Mitrousias. *Writing—review and editing*. Jacques Menetrey and Victoria Duthon. *Visualisation*: Vasileios Mitrousias and Jacques Menetrey. *Supervision*: Jacques Menetrey.

## CONFLICT OF INTEREST STATEMENT

The authors declare no conflicts of interest.

## ETHICS STATEMENT

Obtained by the Commission Cantonale d'Ethique de la Recherche sur l'être humain de Gevève—No. 2020/2865.

## INFORMED CONSENT

Obtained.

## SOCIAL MEDIA HANDLES

Facebook: 1, George A Komnos; 2, Vassilis Mitrousias.

LinkedIn: 1, George A Komnos; 2, Vasileios D Mitrousias; 3, Victoria Duthon; 4, Jacques Menetrey.

## Data Availability

The data that support the findings of this study are not publicly available due to privacy restrictions.
